# Supplementary material for: Clinical and Microbiological Characteristics of Neonates with Candidemia and Impacts of Therapeutic Strategies on the Outcomes
Source: J Fungi (Basel). 2022 Apr 29;8(5):465. doi: 10.3390/jof8050465 (PMC9148079; doi:10.3390/jof8050465)
Supplement: Supplementary file 1 [file jof-08-00465-s001.zip › jof-1675642-supplementary.pdf]

Supplemental Table S1. Univariate and multivariate logistic regression analysis of independent risk factors for clinical treatment failure*

	Univariate analysis			Multivariate analysis		
Variables	Odds ratio	95% CI	P value	Odds ratio	95% CI	P value#
Gestational age						
≤ 27 weeks	1.56	0.69-3.53	0.286			
28-32 weeks	0.42	0.15-1.18	0.101			
≥ 33 weeks	1.0	(reference)				
Underlying chronic comorbidities						
No	1	(reference)		1	(reference)	
One	1.59	0.41-6.24	0.505	1.07	0.24-4.70	0.929
More than one chronic comorbidities	7.44	1.97-28.16	0.003	3.22	0.70-14.72	0.132
Septic shock	3.17	1.49-6.76	0.003	2.10	0.82-5.34	0.120
Delayed CVC removal (> 72 hours)	5.01	2.04-12.35	< 0.001	4.14	1.52-11.23	0.005
Breakthrough candidemia	7.13	2.81-18.06	< 0.001	4.97	1.80-13.76	0.002
Delayed initiation of effective antifungal agents (48 hours)	1.31	0.62-2.76	0.477			
Initial antifungal therapy						
Fluconazole/Voriconazole	1.0	(reference)				
Amphotericin B	0.71	0.30-1.71	0.449			
Echinocandin-based regimen	1.50	0.56-4.02	0.420			
Pathogens						
Candida albicans	1	(reference)				
Non-albicans Candida spp.	1.36	0.66-2.80	0.408			

Uncommon *Candida* spp.**	1.85	0.98-3.49	0.059
Case years			
2003-2006	1.64	0.56-4.82	0.371
2007-2011	2.21	0.81-6.08	0.123
2012-2015	1.56	0.54-4.58	0.416
2016-2020	1	(reference)	

APACHE II: Acute Physiology and Chronic Health Evaluation II score; CI: confidence interval; CVC: central venous catheter

*All-cause mortality within day 3-30 (episodes with antifungal treatment) or persistent bloodstream infection for ≥ 72 hours from the initiation of antifungal therapy in 310 evaluable episodes of *Candida* BSI in children.

#Hosmer-Lemeshow $P = 0.664$

**Uncommon *Candida* spp. included all *Candida* spp. in addition to *C. albicans, C. parapsilosis, C. glabrata, C. tropicalis, and C. krusi*
